# Editorial: Psychological Interventions to Improve Diabetes Self-Management

**DOI:** 10.3389/fcdhc.2022.931125

**Published:** 2022-07-18

**Authors:** Anne M. Doherty

**Affiliations:** Department of Psychiatry, University College Dublin/ Mater University Hospital, Dublin, Ireland

**Keywords:** diabetes - quality of life, diabetes mellitus T1DM, psychological therapies, social support, mental health

Diabetes is an increasingly common condition, and there is frequently a significant burden on the individual with diabetes due to the demands of self-managing a complex long-term condition (1). Many people with diabetes have difficulties in achieving optimal glycaemic control, and psychiatric and psychological factors may present barriers to effective self-management (2). Depression, for example is increasingly being regarded as a risk factor for mortality in diabetes, likely due to the impact of reduced motivation on the active self-management required for good diabetes control (3). Psychological interventions have an important role in the management of suboptimal diabetes care, and there have been a range of studies which have examined various facets of psychological intervention for diabetes optimization (4). There is evidence that specific psychological interventions such as cognitive behaviour therapy (CBT), motivational interviewing and attention control may be effective in reducing diabetes distress and improving diabetes management (5).

Upsher et al. conducted a secondary meta-analysis of a pre-existing systematic review and meta-analysis which had included 67 randomized controlled trials (RCTs). The earlier systematic review and meta-analysis found that adults with type 2 diabetes (T2D) who received a psychological intervention experienced a significant reduction in HbA1c compared with those who did not receive the intervention (5). This secondary analysis was conducted with a view to identifying the “active ingredients” of these psychological interventions, to inform the development of future interventions.

This paper reviewed the psychological interventions in studies included in the pre-existing review and identified the behaviour change techniques (BCTs) utilised in the individual studies. This study identified that ‘social support’ (n=50), ‘problem solving’ (n=38) and ‘goal setting’ (n=30) were the most frequently used BCTs, and all were associated with significant improvements in glycaemic control (HbA1c). On meta-analysis there were no significant associations between HbA1c and type of psychological intervention (p=0.84), or of the frequency of BCTs occurring within the intervention (p=0.29). This study identified that social support, problem solving, and goal setting may have potential in developing future psychological interventions for people with T2D.

McGuigan et al. reported the design of a diabetes-focussed intervention to address psychological barriers to injectable treatments by utilising a behavioural change framework among people with T2D. Approximately 80% of patients discontinue or interrupt injectable regimens soon after commencement: suggesting that this issue is complex, related to broader adherence issues. Poor engagement and adherence are attributed to psychological barriers such as negative perceptions of injectables, depression, anxiety, feelings of shame, distress and perceived lack of control.

The authors based this intervention on a systematic review which identified a need for structured diabetes education focussed on psychological constructs to inform effective interventions to improve the initiation of and persistence with injectable medication for T2D. This systematic review along with findings from focus groups were translated to develop an intervention for people with T2D transitioning to injectable therapies, named *Overcoming and Removing Barriers to Injectable Treatment in T2D* (ORBIT). This intervention comprised identifying the barriers to commencing injectables and pairing these with a broad range of techniques (including education, CBT techniques and utilising supports) to help the patient in overcoming the barriers.

Lowry et al. described a novel intervention called D1 Now which was designed to support self-management and engagement in order to improve outcomes in young adults (18-25 years) living with type 1 diabetes (T1D). It has been developed using a user-centred approach, and incorporating theoretical elements resulting in a three-fold intervention. One of the central components of this intervention is the availability of a Support Worker to provide continuity and build relationships with young adults and their diabetes team.

In this pilot RCT, the Support Worker provided an accessible point of contact for young adults, including conversations about distress, and developing goal setting and collaborative problem solving interventions. Diabetes distress was common in this population and was associated with challenges including cognitive distortions (e.g. ‘all or nothing’ thinking patterns) and disordered eating behaviours. The Support Worker advocated for the young person with T1D with the diabetes team by explaining the challenges and barriers to care elicited in their interactions. This pilot has identified that the role of the Support Worker was viewed positively from the perspective of young adults with T1D.

O’Donnell et al. described the theoretical underpinnings and development of a psychological intervention for parents of young people with T1D aged 11-14. This theoretically informed intervention was designed in recognition of the higher incidence of disordered eating and clinical eating disorders in young people with T1D. Where present comorbid with T1d, disordered eating is associated with negative outcomes in physical and mental health, including repeated diabetic ketoacidosis and hyperglycaemia. There is growing evidence that disordered eating in T1D may be effectively prevented through psychological intervention.

This is a manualised intervention and includes two online group workshops, supplemented by additional online materials intervention was co-developed with an expert advisory group of clinicians, and families of young people with T1D. The findings of the feasibility study will inform the future alignment of this intervention with routine diabetes care. This interventions has the potential to improve the psychological and physical wellbeing of young people with T1D.

## Conclusion

These diverse papers demonstrate the range of psychological interventions which may be utilised to optimise the management of both T1D and T2D, and indicate that different psychological approaches may be required for different challenges, for example a support worker may be effective for the young adult population, but a population with disordered eating may require a more tailored intervention, and specific interventions to address individual-level barriers to commencing injectable therapies may have promise. Four papers do not of course offer a comprehensive overview of the area of psychological therapies in diabetes, but they provide a glimpse of emerging treatment. There is much work to be done in further refining the right intervention for the individual challenges that the person with diabetes may face, and these studies indicate that a person-centred approach to finding solutions to the individual’s specific problems may be approaches which will shape the evidence in the future.

## Author Contributions

The author confirms being the sole contributor of this work and has approved it for publication.

## Conflict of Interest

The author declares that the research was conducted in the absence of any commercial or financial relationships that could be construed as a potential conflict of interest.

## Publisher’s Note

All claims expressed in this article are solely those of the authors and do not necessarily represent those of their affiliated organizations, or those of the publisher, the editors and the reviewers. Any product that may be evaluated in this article, or claim that may be made by its manufacturer, is not guaranteed or endorsed by the publisher.
